# Hospital and Institutional News

**Published:** 1918-02-02

**Authors:** 


### HOSPITAL AND INSTITUTIONAL NEWS.

### THE KING AND THE HOSPITAL SATURDAY FUND.

Lord Cromer has written as follows to Sir Vezey Strong, the Chairman of the Hospital Saturday Fund:—" Dear Sir Vezey Strong,—In Lord Stamfordham's absence I have laid your letter of January 23 before the King, who has read with great interest the details you have been good enough to furnish in regard to the results of the Hospital Saturday Fund Collections during 1917. I am commanded to thank you for enabling His Majesty to follow from your Report the progress that has been made by the Fund, and the King recognises with much satisfaction that the splendid results achieved are mainly due to the unsparing activities of the Honorary Workers, who must indeed be pleased at seeing their efforts crowned with such success.—Believe me, yours very truly, Cromer."

### THE LATE SIR JOHN WOLFE-BARRY, K.C.B.

At the meeting of the House Committee of the Westminister Hospital, held on January 29, the following resolution was passed:—That the committee desires to place on record its sense of the great loss the hospital has sustained by the death of its lamented Chairman, Sir John Wolfe-Barry, who has for twenty years presided over its deliberations with an ability and courtesy which gained the entire confidence and affectionate esteem of his colleagues and carried the affairs of the hospital through many times of difficulty with success." After the above resolution was passed, the meeting, as a mark of respect, immediately adjourned without doing any business.

### A NATIVE LABOUR GENERAL HOSPITAL.

We gave an account of a Native Labour General Hospital somewhere in France in our issue of January 12, page 305. At home we are experiencing the ever-growing necessity for more and more beds, and what is true of combatant hospitals at home is equally true of labour hospitals in France. The O.C. of a labour hospital has special problems, amongst them being that he has to collect and train a native staff from the raw material, and there is no small difficulty attached to the work. To obviate this, native (Chinese) orderlies who show aptitude for ward work are taken on the strength, and in this way Major Frood is able to mould them to the duties of an orderly. But it takes time and patience. Again, the O.C. has had much experience with South African natives, and had a trained staff whom he had brought well up to concert pitch when we visited his hospital. Being, however, under contract, their time of service has since expired, and they have returned to South Africa, where they will be able to carry on this excellent work, as there are many good openings for trained hospital attendants on the mines.

### AN UNIQUE CHRISTMAS CARD.

The demands for native labour have steadily increased, so that the original number of beds in a native hospital has been doubled, and will probably be trebled before many months are out. The Chinese labourers, of whom we saw a great many are young, alert, bright, fine, jovial fellows, who seem to enjoy the work and to take life with commendable cheerfulness. They are often most intelligent, have great skill in design and modelling, and we are the fortunate possessors of a Christmas card, painted and designed by one of them, which we hope to reproduce in The Hospital, unless the colours defeat the photographer and render this impossible. It is extremely ingenious, and represents the unit changed from a South African to a Chinese hospital, which is illustrated by two Chinese dragons swallowing up the springbok, and the R.A.M.C. badge—a red serpent in green surroundings—manifesting itself in protest. " Perfect peace to all below Heaven " in Chinese is written under the name of the hospital, followed by the season's greetings, and the date, 1917, surrounding which is an inscription, again in Chinese, wishing good luck and happiness for the New Year. Separating the gruesome tails of the dragons is written, T. M. Frood, Major R.A.M.C., B.E.F. The card is a gem of its kind, the mouth, eyes, and feet of the dragons, and their tails, being truly appalling to look at and study. It is pleasant to hear that the weather at present is just like spring at the Front, which makes all South Africans feel good. Apart from the general interest which all hospital workers are likely to take in these Native Labour General Hospitals, from their nature and character, those associated with the press can show well-merited appreciation of the good work done—and it is real hard work too—at the isolated and special hospital that we have had the privilege to describe.

### THE LATE SIR G. H. PHILIPSON.

When a veteran full of years but of little else, who has long ceased to be of any professional significance, who indeed may have never had anything to show for himself save a record of pertinacity and self-seeking, dies nowadays it is only too customary to refer to the occurrence as a loss to medicine. But in the case of such a one as the late Sir George Philipson, who passed away rather suddenly on January 24 last, such conventional words are indeed true and real. Lycidas, alas! is dead, and hath not left his peer. A long life of single devotion to medical science has now closed. The erudite works of the Durham professor of medicine, his encyclopædic knowledge of the world's literature of his subject, his mastery of every latest development, have long been the envy of younger colleagues while his frankness, his generosity of mind, unselfishness, scorn of " the arts by which men rise," won him that respect from practitioners, and particularly students, which was so evident and well known. Newcastle-upon-Tyne, the home of Stephenson, Armstrong, Eldon, and that other poet-physician Mark Akenside, must now again mourn an illustrious son departed; but whereas not all of these earlier names are commemorated by local memorials, Sir George Philipson's impressive features persist in several forms of plastic art. A fresco painting on the walls of one of the medical buildings in the city is particularly noteworthy for its portrayal of his straightforward and keen but kindly glance and the native dignity of his bearing.

### THE NEED FOR EXTENSION AT COVENTRY.

COVENTRY is one of those towns the population of which has increased during the war, and therefore it is satisfactory to see that the hospital has paid its way during the past year. The only debt is on the extension fund account. The workers' contributions have increased by £1,000, and their subscriptions have practically doubled since 1914. Apart from this, and the War Office payments, the income has grown by about £3,000. In fact, if the cost of commodities had not advanced so much, the cost per bed would have fallen from £82 to £62. There is, however, an urgent need for extension, and the hospital at present is somewhat crowded. The position, indicated by the waiting-list, is becoming acute, and before long it will be a grave one. Mr. E. M. Iliffe was in his place again at the annual meeting, after an absence of three years, previous to which he was in regular weekly attendance at the board meetings. Miss Hutchinson, who was appointed matron some three years ago, was warmly praised for her economical management, to which the relative fall in the cost per bed was mainly attributed. If the hospital does not fall behind its work its extension must be decided on, and its present financial position shows that more support must be forthcoming if it is to shoulder the proper burden which the population has placed upon it.

### WAR DIET AND PUBLIC HEALTH.

LIEUT.-COL. CHARLES BROOK, R.A.M.C., in presiding at the annual meeting of the Lincoln General Dispensary, attributed the good health of the city during the past year to the scarcity of food and its beneficial effect upon the population. There had been, he said, no increase in the illness or epidemics in the city, which he attributed to the larger abstention from alcohol and the dearness and scarcity of food. Less time was spent in eating and drinking than before. The factors which produce and maintain public health are so complex that it is dangerous to make ready guesses at their cause, and it will be admitted that the pinch in England is only beginning to be felt, and that till last autumn it was more apparent than real. In France tobacco can be obtained once a week only, the sugar ration is one pound per month, and during the winter the coal ration has been three cwt. a month only. It is difficult to gather statistics from other countries, and still more to mark the precise point at which food scarcity amounts to under-feeding. Besides, the reduced consumption of alcohol has its reverse side in leading to home drunkenness, which has to be recorded of certain districts. But it is safe to say that we have felt the pinch in England less and later than any belligerent except, for the present, America.

### THE GARRETT ANDERSON MEMORIAL.

IT has been decided that the memorial to Dr. Elizabeth Garrett Anderson shall take the form of endowing fifty beds in the New Hospital for Women which she founded. To do this £50,000 will have to be raised, and an enthusiastic start has been made by the various women's colleges. The "Oxford" bed is being endowed by Somerville, Lady Margaret Hall, and St. Hugh's College, which are busy collecting funds. Girton and Newnham are doing the same for a "Cambridge" bed, and other beds are being endowed by Bedford College and Queen's College. Nor does this exhaust the opening list. Women in the various professions open to them, and men sympathisers also, are banding together. The difficulty is probably that more women are reaping the benefit of Dr. Garrett Anderson's work than realise how much their opportunities owe, directly or indirectly, to the force of her example. The number of women engaged on the occupations touched by colleges and institutions is still relatively small compared to the number of women workers. For instance, the nursing profession might well be expected to endow a bed at the New Hospital, but do its many members feel a natural sympathy with the aims for which Dr. Garrett Anderson fought, and which the New Hospital has done so much to bring to fruition?

### A VERY RARE DISTINCTION.

SOME interesting details were brought out at the recent half-yearly meeting of the governors of the East Suffolk and Ipswich Hospital. While the average number of occupied beds has reached the record figure of 366, a remarkable fact is the virtual absence of a waiting-list. In spite of an influx of women patients, to whom the larger upper ward has been allotted, the number of women waiting admission is nine only; "the children's list has been entirely cleared," and the number of men on the waiting-list is reckoned at eight. This is a most unusual state of affairs, and should do more than anything to attach the interest of the town and district to the hospital. For how can people tell that a hospital is "theirs" if an immense waiting-list stands between them and a chance of admission? It is only of late that the public conscience has been stirred in this matter, and it is a welcome change to be able to point to an important hospital where a waiting-list is not to be found. We heartily congratulate the board and Mr. Arthur Griffiths, the secretary, upon this rare achievement. Mr. J. D. Cobbold must be, in this respect, the least anxious of hospital chairmen.

### FREE BREAD FOR BELFAST HOSPITALS.

THE wounded in the Belfast hospitals are to benefit by two schemes which concern the establishment of curative workshops and a free supply of bread. According to the correspondent of the *Morning Post*, these workshops are for the Ulster Volunteer Force Hospital, while a plan is on foot to supply free bread to the six hospitals organised by the Ulster Volunteer Force, which accommodate 604 beds. The bread scheme, which is expected to save the hospitals £1,200 a year, was started at the municipal institute, where the bread will be baked at the existing model bakery. A joint committee of master bakers and members of the Bakers' Trade Union is in charge of the arrangements. The master bakers and the flour merchants will provide the materials, and the men will take turns at the bakery. To these gifts is to be added by the Corporation the free use of the bakery and the fuel, and by these means the heavy expenses incurred in baking for the hospitals will be saved.

### THE FATE OF AN ART GALLERY.

THE Hull Corporation Property Committee has been considering the terms under which the city council is prepared to hand over the city art gallery to the Recruiting Medical Board. The Office of Works has proposed that no rent should be paid by the Ministry of National Service to the corporation, although a rent of £300 a year had been suggested. It was urged by one speaker that Hull was doing more than its share in being asked to take over the recruiting work of the East Riding. If other centres in the Riding had been made to do their proper share Hull would not have been called upon to surrender its beautiful art gallery. The chairman of the committee, Sir Alfred Gelder, held that a great mistake had been made in making the offer to the Ministry, for it implied, quite wrongly, that there was no use for the gallery. If it were commandeered, and not conceded voluntarily, the committee could go before the Losses Commission to prove their out-of-pocket expenses and any damage which the building received. The chairman, the town clerk, and a number of the committee were deputed to discuss the situation with the Commissioner of Works and to explain how the town had been treated in regard to the city hall. A Recruiting Medical Board would be elaborately housed in any city art gallery, and we are surprised that a city like Hull should have offered its art gallery for this purpose.

### CHARITABLE DONATIONS AND THE EXCESS PROFITS TAX.

A QUESTION which is exercising the minds of some of those who are fortunate enough to have to pay excess profits duty is as to whether deductions may be allowed in respect of charitable donations. The responsible task of collecting the taxes due in the City of London is confided to a number of tax surveyors in Telegraph Street, and one of these has allowed a rebate in respect of donations to war charities on the following grounds: that excess profits duty would not be imposed were it not for the war; that war charities owe their existence to the same contingency; and that therefore one factor may equitably be considered in relation to the other. It is obvious that a rebate from the sum assessed as excess profits may mean a considerable saving to the payer, and the point is well worth the attention of individuals and firms doing war work. Hospitals which are treating wounded soldiers have an obvious interest in the matter, and secretaries should not fail to impress upon rich firms the double advantage to be thus gained; for not merely will charitable donations bring their usual meed of moral blessing, but they may produce a not-far-distant reward of a pecuniary nature; while the especial virtue of the proposition is, that the larger the donations given, the greater will be the advantages accruing to both parties.

### ENDOWED BED FOR AN ATHLETE.

IT not infrequently happens that a bed is founded in a hospital for the use of members of a particular religion, class, or profession. Necessarily this does not involve a particular bed being kept available and waiting for a case within the meaning of the terms of endowment, but merely that so far as is possible the bed shall be used for such a case. Particularly must this be so where the profession selected is one with comparatively few members, as is the case in respect of an endowment by the late Mr. Richard Smith, of the Trees Inn, Bristol Road, Birmingham, by means of a legacy of £1,250 to the General Hospital, of a bed for the preferential use of any persons who are, or shall at any time have become, engaged as professional gymnasts, acrobats, or athletes. Curiously, the word "gymnast" conveys quite a different meaning in a hospital sense from that probably in the mind of Mr. Smith when he made his will; he most likely did not know of the gentleman, generally of Swedish extraction, who conducts the treatment in our exercise wards, and who, in a strictly legal sense, can now in time of necessity make claim upon the ward accommodation at the General Hospital, Birmingham.

### WAR BONDS AND HOSPITALS.

WITH War Bond Tanks wandering about the country there is a splendid opportunity for the generous-minded to help their country and help their own particular hospital at one and the same time. The way in which this may be done has been shown in Nottingham and Swansea. In the latter town Miss Dulcie Vivian, of Clyne Castle, has sent to Mr. Joseph Hall, secretary of the Glynn Vivian Home of Rest for the Blind at Mumbles, a cheque for £100, asking him to invest it in a War Bond at the Tank at Swansea as a donation towards the endowment of the Home. At Nottingham Sir Frank Bowden, Bart., marked "Tank Week" by not only investing a goodly sum in National War Bonds on his own account, but generously putting in £2,000 as a special donation from him to the Nottingham General Hospital. The Tanks are still on tour from one provincial town to another, and these good leads may well be followed by wealthy, kindly-disposed individuals elsewhere, to the benefit of their respective hospitals. Such gifts have a double usefulness—they help the country in its hour of financial need and they help the hospitals, whose needs are always clamant.

### WOMEN AS SANATORIA SUPERINTENDENTS.

The Health Committee of the Southend Corporation have reported on the visits of inspection made by a special sub-committee and Dr. Banister, the medical officer of health to the Merivale and the Maltings Farm, Nayland, Sanatoria, concerning both of which complaints had been received from the Southend Insurance Committee. The brief summary of the report which has come to our notice illustrates very well a point in sanatorium management often stressed in the columns of The Hospital. It is true that the accommodation at Maltings Sanatorium, as to which no complaints had been received (except from the Southend Insurance Committee—but then Southend has been the scene of other medical quarrels than this) is of a more substantial nature than that at Merivale. But what experience detects as the real cause is another difference between the two institutions. The one is under male management, the other under female. Even if the Merivale consumptives are given a recreation-room, it is to be feared that that will not content them for long. Their complaints as investigated by their own friends turn out to be largely groundless. The one about the drainage is proved to be sheer imagination. Those who know sanatorium life from the inside know that men will not obey women. Nothing else than that ruined a private sanatorium, prominent some years ago, now given up. The patients said they were willing to obey the doctor but not his wife. Nothing else than this is responsible for half the cases of tuberculous soldiers having enough of sanatorium matrons and leaving before their health is in any way restored. Disobedient patients, contumacious patients, are always grumblers; and always those from the poorest homes—perhaps from the workhouse—turn up their nose at the food. Half a dozen sharp, plain words from a male medical officer who takes his meals with the patients, eating the same food as they do, would have cleared the "atmosphere" spoken of at Merivale like magic. But a woman cannot do this. Nor would it be any good if she could. For a virago is felt to be unnatural, and resented accordingly. At bottom, then, the trouble is a war trouble. The measures prescribed will be of temporary benefit. But a radical cure is not likely until Dr. Marrett returns, or a man of keenness and determination is put in his place.

### OPHTHALMIA NEONATORUM.

The Metropolitan Asylums Board have purchased from the Guardians of St. Pancras St. Margaret's Home and some adjacent property in Kentish Town for the purpose of establishing a hospital for the treatment of ophthalmia neonatorum, and later the Board proposes to establish a similar hospital in the South of London. When it is borne in mind that the vast majority of cases of blindness (apart from those cases arising in the war) are due to that form of ophthalmia which occurs in the newly born, it is seen at once how important for the individual and for the community at large it is to prevent as many of these cases as possible, and it is only in a suitable institution that the best results can be obtained. There is no room for most of them in the ophthalmic hospitals. By early and thorough treatment it is possible to save every case.

### THE PRISONERS' NEW PAPER.

The number of war publications dealing with the work of different authorities and institutions continues to grow, and the latest is the *British Prisoner of War*, which made its first appearance in January as the monthly journal of the Central Prisoners of War Committee of the Red Cross and Order of St. John. The life of prisoners' camps and information on the regulations governing prisoners of war, accompanied by maps and illustrations, form its staple subject. Care Committees will now have their own paper for the exchange of news, and the public can hardly fail to appreciate the information. In the first number occurs a copy of the sugar permit and a map showing the whereabouts of the principal prisoners' camps in Germany. It is published by Messrs. Nisbet, of 22 Berners Street, price one shilling. In Turkey, we learn from an interesting article that British and Indian prisoners who are fit to work are engaged on railway construction. Such questions as what and how to send to prisoners in neutral countries are answered, and the reason why few acknowledgments of parcels are received from prisoners in Turkey is explained. When the paper gets into its stride it should be even more interesting than at present.

### THIS WEEK'S DRUG MARKET.

Several noteworthy advances in prices have taken place this week. Following on a rise in the value of bismuth metal, makers of bismuth carbonate and bismuth subnitrate and other salts have raised their quotations. English refined camphor is dearer, and in view of the shortage of supplies a further rise in price is not improbable. Sugar of milk is scarcer than ever and substantially dearer. Higher prices are asked for cascara sagrada and senega root. Benzoic acid and sodium benzoate are dearer. The shortage of bromides is as acute as ever, and a further advance in makers' quotations is not unlikely. Very little glucose appears to be available. Makers of chloroform and ether find it difficult to meet the large demand. A considerable quantity of Russian henbane has arrived, and this may relieve the market to some extent. Exports of cod-liver oil from Norway have been prohibited by the Norwegian Government owing to the shortage of fats in that country. Salicylic acid and sodium salicylate have a slightly upward tendency in price, but at present there is no quotable change. A further small business has been done in quinine, and the value is very firmly maintained.

